# Fifteen years of sector-wide approach (SWAp) in Bangladesh health sector: an assessment of progress

**DOI:** 10.1093/heapol/czv108

**Published:** 2015-11-18

**Authors:** Karar Zunaid Ahsan, Peter Kim Streatfield, Rashida -E- Ijdi, Gabriela Maria Escudero, Abdul Waheed Khan, M M Reza

**Affiliations:** ^1^MEASURE Evaluation, Carolina Population Center, the University of North Carolina at Chapel Hill, NC-27517, USA,; ^2^ICDDR,B, Shaheed Tajuddin Ahmed Sharani, Mohakhali, Dhaka 1212, Bangladesh and; ^3^Program Management & Monitoring Unit, Ministry of Health & Family Welfare, Dhaka-1000, Bangladesh

**Keywords:** Bangladesh, development partners, health policy, health reform, sector-wide approach

## Abstract

The Ministry of Health and Family Welfare (MOHFW) of the Government of Bangladesh embarked on a sector-wide approach (SWAp) modality for the health, nutrition and population (HNP) sector in 1998. This programmatic shift initiated a different set of planning disciplines and practices along with institutional changes in the MOHFW. Over the years, the SWAp modality has evolved in Bangladesh as the MOHFW has learnt from its implementation and refined the program design. This article explores the progress made, both in terms of achievement of health outcomes and systems strengthening results, since the implementation of the SWAp for Bangladesh’s health sector. Secondary analyses of survey data from 1993 to 2011 as well as a literature review of published and grey literature on health SWAp in Bangladesh was conducted for this assessment. Results of the assessment indicate that the MOHFW made substantial progress in health outcomes and health systems strengthening. SWAps facilitated the alignment of funding and technical support around national priorities, and improved the government’s role in program design as well as in implementation and development partner coordination. Notable systemic improvements have taken place in the country systems with regards to monitoring and evaluation, procurement and service provision, which have improved functionality of health facilities to provide essential care. Implementation of the SWAp has, therefore, contributed to an accelerated improvement in key health outcomes in Bangladesh over the last 15 years. The health SWAp in Bangladesh offers an example of a successful adaptation of such an approach in a complex administrative structure. Based on the lessons learned from SWAp implementation in Bangladesh, the MOHFW needs to play a stronger stewardship and regulatory role to reap the full benefits of a SWAp in its subsequent programming.

Key Messages

- Since its inception, the SWAp modality in Bangladesh’s health, nutrition and population (HNP) sector has evolved as the Ministry of Health and Family Welfare (MOHFW) has learnt from its implementation and refined the program design.
- SWAp contributed to an accelerated improvement in key HNP outcomes in Bangladesh over the last decade.
- Hybrid nature of SWAp in Bangladesh allowed the MOHFW to continue implementing development projects outside the SWAp, diverting resources.
- Due to overly centralized planning processes inherent to the government system, MOHFW may focus on strengthening its stewardship and regulatory role to reap the full benefits of a sector-wide approach.

## Introduction

### Sectoral context

The Ministry of Health and Family Welfare (MOHFW) is responsible for the formulation, implementation, management, coordination, and regulation of national health, nutrition and population (HNP) related activities, programmes and policies. In line with the general system of public administration in Bangladesh, the MOHFW management structure comprises the Secretariat, responsible for policy development and administration comprising eight functional wings and units, and the Directorate General of Health Services (DGHS) and the Directorate General of Family Planning (DGFP), which are responsible for implementation of HNP services in the field. Apart from these two, several other Directorates (e.g. Directorate of Nursing Services, Directorate of Drug Administration, etc.) perform designated administrative duties under MOHFW.

Following independence in 1971, development partners (DPs) played a key role in supporting the Government of Bangladesh (GOB) through financing a series of projects focused on family planning (FP) and health. Table 1 summarizes the major HNP projects in Bangladesh since independence (World Bank 1998; White 2007).

**Table 1.** Major HNP-related projects in Bangladesh, 1975–98

Project name	Duration	Objective	Financiers
Bangladesh First Population Project	1975–80	Increase use of FP and MCH services	World Bank, Australia, Canada, Germany, Netherlands, Norway, Sweden and United Kingdom
Bangladesh Second Population and Family Health Project	1980–86	Development of national FP programme	World Bank, Australia, Canada, Germany, Netherlands, Norway, Sweden and United Kingdom
Bangladesh Third Population and Family Welfare Project	1986–91	Reduction of fertility and IMR	World Bank, Australia, Canada, Germany, Netherlands, Norway and United Kingdom
Bangladesh Fourth Population and Health Project	1992–98	Reduction of fertility and IMR, improvement of MCH	World Bank, Australia, Canada, Germany, Netherlands, Norway, Sweden, United Kingdom and European Union.

These projects were targeted separately for health and FP, which was neither efficient nor sustainable (World Bank 1998; Simpson *et al*. 2001). The MOHFW recognized that the existing health system was not suited to deliver cost-effective and integrated health services (Martinez 2008). The MOHFW envisaged that a sector-wide approach (SWAp) would meet the identified challenges in three ways: (1) improved coverage of essential health and FP services would be assured through technical support and coordinated financing; (2) service delivery would be more cost-effective by leveraging sector reforms and (3) involvement of NGOs and the private sector in service delivery would be promoted (World Bank 1998). These aspirations provided the impetus for the MOHFW to embark on a sector-wide development programme.

### SWAp in Bangladesh’s health sector

The Health and Population Sector Strategy (HPSS) of 1997, prepared jointly by the GOB and DPs starting in late 1995, marked the decision to move away from a project-based modality to a SWAp. Under the HPSS, the GOB agreed to implement the health programme through operational plans (OP), each led by a Line Director (LD). Each OP included a set of SWAp activities along with budgets, and periodic reviews of performance and resources led by the MOHFW. At the end of each year, the GOB agreed to annual program reviews (APR) focused on programme implementation and actual expenditures conducted by a team of independent experts.

The first SWAp was the Health and Population Sector Programme (HPSP) 1998–2003. It was led by the government and funded by the Government and DPs with pooled and bilateral funding. The main focus of the HPSP was to decentralize the delivery of the essential service package (ESP) of primary health care (PHC) using a ‘one-stop’ service model, to deliver basic health and FP services to rural communities from static community-based Community Clinics (CC). In 2003, the second SWAp, titled the Bangladesh Health, Nutrition and Population Sector Programme (HNPSP) was designed and implemented during 2003–11. The overall objective of the HNPSP was to increase the availability and utilization of user-centered, effective, efficient, equitable, affordable and accessible quality HNP services. Based on the successes and lessons learned in the previous SWAps, the MOHFW adopted the third SWAp in 2011 entitled the Health, Population and Nutrition Sector Development Programme (HPNSDP) 2011–16. The focus of HPNSDP is to strengthen health systems and improve health and FP services. Table 2 below illustrates the duration, size and the government’s contribution to SWAp financing of the successive SWAps in Bangladesh’s health sector (World Bank 2005a, 2011, 2012b). The financial contribution of GOB to the health SWAp has risen over the last 15 years while the proportionate share from the DPs has gradually been falling.

**Table 2.** Health SWAps in Bangladesh, 1998–2014

Programme name	Duration	Fund (GOB contribution)	Co-financiers
Health and Population Sector Programme (HPSP)	1998–2003	US$ 2.2 billion (62%)	World Bank, Canada, Germany, Netherlands, Sweden, United Kingdom and European Union
Health, Nutrition and Population Sector Programme (HNPSP)	2003–11	US$ 5.4 billion (67%)	World Bank, Canada, Germany, Netherlands, Sweden, United Kingdom, European Union and UNFPA
Health, Population and Nutrition Sector Development Programme (HPNSDP)	2011–16	US$ 7.7 billion (76%)	World Bank, Canada, Sweden, Australia, United Kingdom, Germany and United States.

The overarching objective of all three HNP SWAps has been to improve access to and utilization of an essential package of health, population and nutrition services, particularly by the vulnerable population groups, viz. poor women and children. (World Bank 1998; GOB 2005, 2011b). The health SWAps in Bangladesh are all characterized by a) a predominant focus on maternal and child health (MCH) and FP services and b) a boundary of other health activities implemented within the health sector but which do not fall under the MOHFW SWAp, though the SWAps are designed considering the entire health sector planning. For example, Bangladesh health SWAps have not included the public sector health interventions implemented outside the MOHFW (e.g. urban heath programme of the Ministry of Local Government, health programmes of the Ministry of Social Welfare, etc.), nor the health programmes implemented by NGOs or by the private sector. In fact, there also exist large projects within the MOHFW (e.g. construction of tertiary hospitals), which fall outside the purview of the health SWAp.

Recognizing that in a sector-wide programme, monitoring is crucial to assess progress of programme implementation, correct problems and inform the design of the next-year programme, the GOB has put emphasis on monitoring and developing indicators and a set of standards to be met. The programme performance indicators are agreed upon by the MOHFW and DPs, and revisited each year. This has led to changes in programme design, planning and monitoring practices in the HNP sector, making it different from the project approach followed in other sectors of the economy.

### Objective of the assessment

Despite the popularity of SWAp among the DPs as the preferred way of providing development assistance (Garner *et al.* 2000), there is scant evidence in the scientific literature that the approach is well implemented or effective, particularly in the health sector (Negin 2010). Against this backdrop, this article reviews the progress made under the successive HNP SWAps in Bangladesh since 1998, and documents whether and how far the SWAp has a) strengthened selected health systems components to effectively provide services by the MOHFW and b) improved service access to the citizens of Bangladesh, which consequently improved the overall health status of the country. In order to demonstrate results for health service utilization and health outcomes, this article focuses on fertility and maternal health, which received the MOHFW’s main attention (and the majority of investment) during the SWAp period.

## Methods

Due to the design and scope of implementation, assessing the impact of a SWAp poses particular methodological challenges, particularly in contexts like Bangladesh where a strong presence of private sector and DP-led parallel programmes implemented by NGOs co-exist with the SWAp. In the absence of key requirements of an impact evaluation design (baseline, counterfactual), this article attempts to provide a brief account of progresses made, or lack thereof, in key SWAp elements along with planned goals in Bangladesh’s health sector using secondary data and available literature. For this assessment, the key elements for Bangladesh health SWAp are considered as: a) an agreed health sector plan; b) government ownership; c) partnership between DPs and government; d) increased funding availability and longer term commitment; e) effort to streamline funding arrangemnts; f) institutional capacity and good governance and g) stability of DP and government personnel (Negin 2010).

This assessment is primarily based on (i) a desk review of existing national and international literature on modalities, aid effectiveness, planning and financing, and monitoring and evaluation of Bangladesh health SWAp; and (ii) review of trend data from periodic, nationally representative cross-sectional surveys and the MOHFW’s financial reports. In order to compare the trends in selected health output and outcome indicators, data from six rounds of Bangladesh Demographic and Health Survey (BDHS) were used, roughly covering the period 1991–2010. Data from two rounds of the Bangladesh Maternal Mortality Survey (BMMS, 2001 and 2010 rounds) and the Bangladesh Health Facility Survey (BHFS, 1998 and 2011 rounds) were also used to compare selected indicators between pre-SWAp and SWAp periods.

All BDHS reports provide nationally representative data on population, nutrition and MCH. Detailed descriptions of the study designs are available in the country-specific reports (NIPORT *et al*. 2013; ICF International 2014). BMMS are high-quality and highly comparable household surveys, designed to assess the situation of the country with respect to maternal health and mortality. Both BMMS are large, covering 99 202 households in 2001 and 168 629 households in 2010, and are nationally representative using a three-stage sampling design (NIPORT *et al*. 2003, 2012). The BHFS collected data from nationally representative random samples of primary- and secondary-level public sector facilities to assess the provision of selected health services (Rannan-Eliya and Somanathan 1999; World Bank 2012a).

## Results

### Implications of SWAp in Bangladesh’s Health Sector

#### Programme management and financing

The SWAp initiated in 1998 replaced 128 discrete projects in the MOHFW (GOB 2003)—this marked a shift towards a more integrated, better-planned delivery of HNP services in Bangladesh (Arifeen *et al*. 2014). MOHFW ownership and leadership were relatively weak in the early years of the SWAp, which were reflected in a) management of SWAp’s APR by the DP-supported and World Bank-run Programme Support Office (PSO) (IEG 2006; Vaillancourt 2009); and b) DPs’ reluctance to relinquish control over the aid management and coordination due to ‘weak government capacity, inadequate accountability and compromised integrity’ (Buse 1999; Negin 2010).

However, the MOHFW’s role in sector coordination and management strengthened over time, as envisioned in the Programme Implementation Plan of HPSP and the follow-on SWAps through detailed capacity building. Within the first few years of HPSP, significant progress in developing the MOHFW’s management capacity in the Directorates and at upazila-level was observed (Simpson *et al*. 2001). The gradual improvement in management capacity of the MOHFW is also evidenced by the strong presence and performance of the MOHFW during the 2008 mid-term review (MTR) of HNPSP (Martin 2009; Vaillancourt 2009). The 2012 and 2013 APRs and 2014 MTR were functionally managed by the MOHFW (GOB 2013a, 2014c).

**Figure 1.**
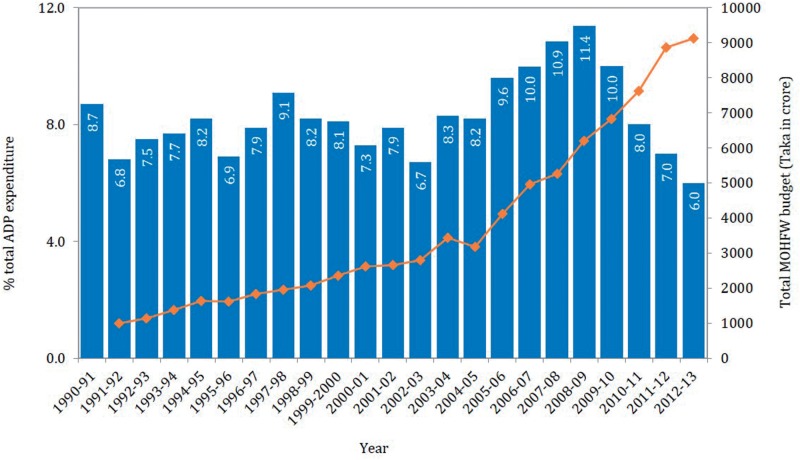
GOB’s ADP Expenditure for MOHFW, 1991–2013 (World Bank 2005a, World Bank 2011, World Bank 2012b)

In terms of financing for the SWAp, the total budgetary allocation for the MOHFW increased at a much higher rate during the SWAp period (24% increase per annum during 1998–2013 compared with 16% during 1992–1998) (GOB 2001, 2006, 2010, 2013b, 2014a). The share of the GOB’s annual development plan (ADP) allocation for MOHFW remained 9% and below during 1991–98, which increased to 11% till 2009 (Mitra 2008), and steadily decreased thereafter to 6% in 2013 (GOB 2014c). Despite considerable increase of financing in absolute figures, this reduction in share was largely due to the incremental size of the national ADP allocation and bulky investments in energy sector as well as large infrastructure projects such as the ‘Padma bridge’ into the GOB’s development budget (Figure 1).

#### Policy formulation and planning

The development planning in Bangladesh historically involves preparation of five year plans (FYPs) and its implementation through yearly budgetary allocations against each development project. Following this practice, a series of FYPs guided the GOB’s policy directions in the health sector during the pre-SWAp period (Mridha *et al*. 2009). Major policy and programmatic steps during the late ‘80 s focused on delivery of core MCH services to rural population through secondary and primary-level facilities and field-level workers. Later in the 1990s, GOB initiated an Emergency Obstetric Care (EmOC) programme that focused on upgrading existing government facilities in phases with specific support from DPs [UNICEF to strengthen District Hospitals and selected Upazila Health Complexes (UHCs), UNFPA to strengthen Maternal and Child Welfare Centers (MCWCs)].

**Figure 2.**
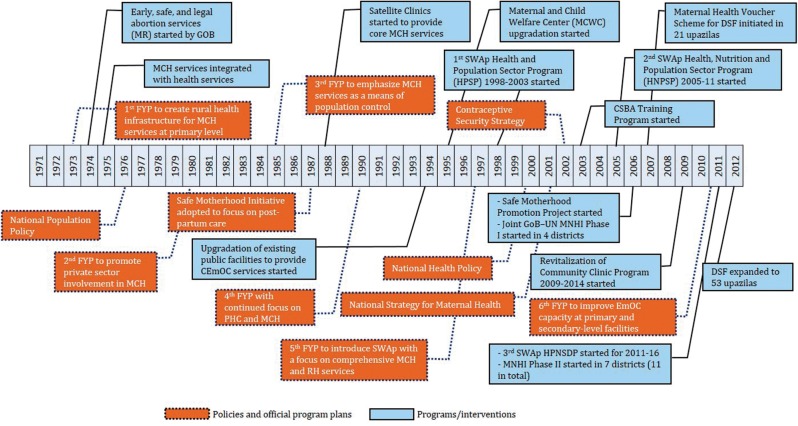
Timeline of major policy formulation and programme initiatives relevant to MCH in Bangladesh

With HPSP, the focus of health service delivery was realigned from home-based service delivery to the provision of services from fixed site clinics and the expansion of EmOC services from health facilities (Rahman *et al.* 2003). The Bangladesh National Strategy for Maternal Health 2001 laid down a detailed, theoretical framework with budgets to improve maternal health in the country, which guided subsequent SWAps since 2003 (Arifeen *et al*. 2014). Each SWAp Strategic Plan outlined specific interventions directly linked to resources within designated OPs—this ensured actual delivery of MCH and other planned services under the SWAp. The policy timeline in Figure 2 (adapted from Arifeen *et al*. 2014) illustrates that a better balance between policy and programmes (for this analysis, we focused on MCH services only) was achieved during the SWAp period.

#### Reform initiatives

Improving health sector efficiency through innovations and policy as well as institutional reforms has been a focused area under the SWAps. Major policy reform areas identified in the successive strategic plans include introducing an ESP, diversification of services by involving stakeholders including NGOs, and review and development of various policies and strategies (e.g. gender strategy; drug policy; etc.). Major institutional reforms tried under the SWAps include integration of services provided through two different Directorates, decentralization, improving financial management (FM) and procurement systems, outsourcing of services and setting up NGO contracting system. (World Bank 1998, 2005b, 2011).

There have been mixed results in achieving planned reforms during the SWAps in the health sector—reforms like integration of health and FP services, diversification of service delivery and modernization of the health sector through decentralization and local level planning (LLP) were not seriously pursued. On the other hand, improvements in programme implementation, strengthening of M&E, procurement and FM processes have registered notable successes (Daniels and Kabir 2014; IEG 2014).

A number of reform initiatives during the first two SWAps were unsuccessful despite strong DP persistence primarily due to insufficient adaptation of the planned activities to the changing policy environment. To overcome this limitation, a matrix of policy issues was developed by the DPs during HPNSDP preparation to engage the MOHFW in a constructive dialogue to ensure MOHFW’s ownership and commitment to these reforms. During the first 3 years of HPNSDP, notable progress has been made in 6 out of 11 major reform activities highlighted in the policy matrix. These include: allocating resources for scaling up EmOC and FP services in lagging regions; mainstreaming nutrition; improving coordination between DGHS and DGFP; developing health care financing policy position paper; revitalizing CCs and strengthening the fiduciary capacity of the MOHFW (GOB 2014b). Also, a coordinated technical assistance (TA) approach has been put in place supported by a multi-year, integrated and consolidated TA plan to mobilize resources in support of selected reforms (World Bank 2011).

During 1998–2013, a number of new initiatives were introduced under the SWAps to help bring systems improvement to accelerate HNP services in the public sector. A list of selected health systems reforms that have taken place since the beginning of the SWAp include:

- Successful revitalization of CCs, which was originally initiated in 1998 to cover around 6000 people in rural areas each with institutional scope for participation by the community representatives in its management. CCs have become popular among rural populations for management of general illnesses, as evident from the growing number of service recipients (mostly women and children) over time—from 12 persons per CC per day in 2009 to 38 persons per CC per day in 2013 (DGHS 2012; GOB 2014b);
- For stimulating demand for basic health services such as delivery care, MOHFW initiated a voucher scheme to enable poor pregnant women to purchase maternal health services under a demand-side financing (DSF) modality. DSF was initially piloted in 21 upazilas (out of 488 upazilas in total) and gradually expanded to 53 during HNPSP (World Bank 2012b). DSF has been successful in increasing skilled delivery and substantially increasing safe motherhood practices in the pilot areas, and significantly increased facility delivery compared with non-DSF areas (HERA 2013). However, during the first three years of HPNSDP (2011–14), planned scale up of DSF did not take place due to strong reservations from major SWAp co-financiers on the ground of financial viability and unattended management shortfalls (GOB 2014b);
- Mainstreaming nutrition through the existing service delivery arrangement in the DGHS and DGFP, in place of vertical, geographically targeted nutrition interventions implemented through NGOs (contracted by MOHFW) has been a major step under the ongoing SWAp. Mainstreamed nutrition services are provided by the newly introduced PHC service providers in the CCs and the focus is on coordinating service delivery and incorporating nutrition data among related OPs;
- Based on lessons learned from previous SWAps, two new institutions—the Programme Management and Monitoring Unit (PMMU) and the Procurement and Logistics Monitoring Cell (PLMC)—were established to strengthen critical aspects of the programme such as management and monitoring, and procurement respectively. During the first 3 years of HPNSDP, both the institutions were found to be contributing to higher efficiency and achieving better results (GOB 2014b).

#### Provision of health services

**Figure 3.**
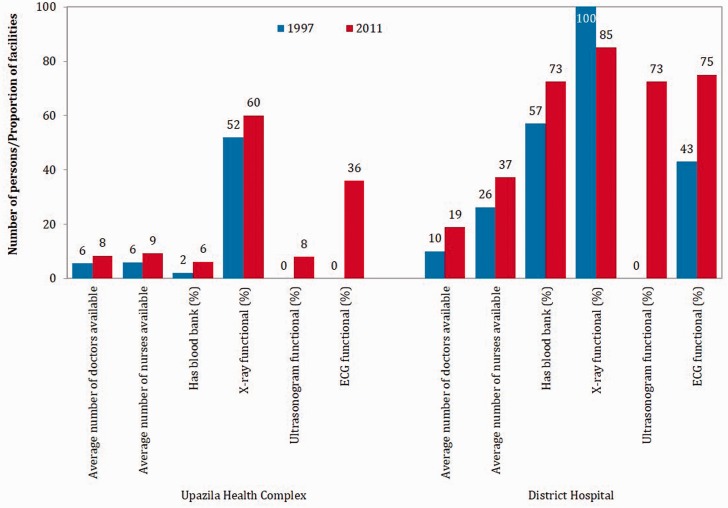
Selected indicators on service provision in primary- and secondary-level government health facilities, 1997 and 2011

**Figure 4.**
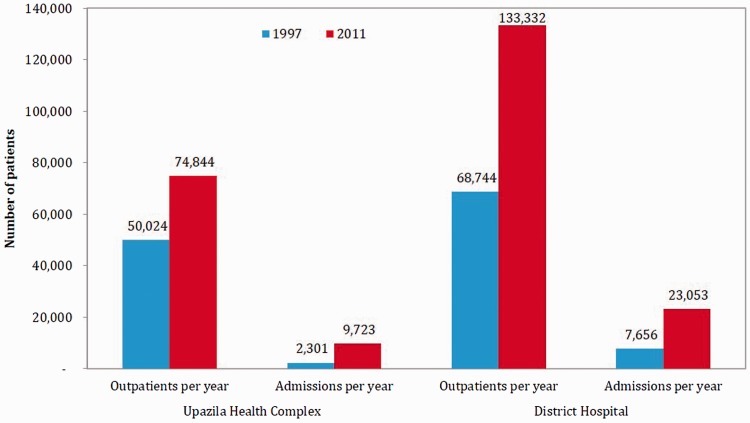
Selected indicators on service outputs in primary- and secondary-level government health facilities, 1997 and 2011

Comparison of selected health facility statistics between 1997 and 2011 indicate that service provision improved in both primary (viz. UHC) and secondary (viz. District Hospital) level facilities. Between 1997 and 2011, availability of physicians, nurses and functional equipment improved under the health SWAp (Figure 3), which helps to explain the substantial increase in outpatient consultations and admissions in government health facilities (Figure 4). During 1997–2010, the hospital beds in public hospitals increased by 51% (from 29 106 to 43 996) (GOB 2010)—more than double the rate of increase in total population during the period (22%) (UN 2013). In terms of hospital bed/population ratio, there were 5350 population for each public hospital bed in 1981, which dropped to 4293 in 1997 and to 3435 in 2010 (GOB 2010; UN 2013).

#### Health service utilization

**Figure 5.**
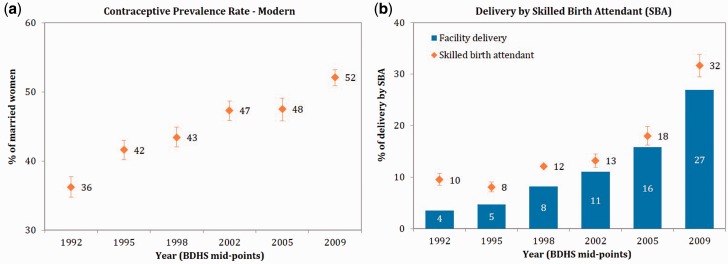
Trends in selected service utilization indicators in Bangladesh, 1992–2009

Contraceptive prevalence rate (CPR) among married women in Bangladesh increased from 8% in 1975 to 61% in 2011—and CPR for modern methods increased from 5% to 52% during this period (NIPORT *et al*. 2013). Despite a nationwide stockout of injectables in 2007 (Al-Sabir *et al*. 2009), use of modern contraceptives increased by 1.7% annually during the SWAp period (1999/2000–2011). However, during the pre-SWAp period (1993–1999/2000), CPR for modern methods increased at a faster rate (2.8% increase annually) (see Figure 5a). In this case, use of contraceptives continued to increase during the SWAp period, but at a slower rate.

In terms of maternal health services utilization, delivery by a medically trained provider increased from 10% to 32% during 1992–2009, mostly driven by an increase in facility delivery during this period (see Figure 5b). The latest round of surveys on Utilization of Essential Service Delivery (UESD) showed that delivery by a medically trained provider in Bangladesh continued to increase and reached 34% in 2013 (Sultana *et al.* 2014). Facility delivery increased from <4% to 27% during 1992–2009, and by 1.6% points every year after 1998 (compared with 0.5% points during 1992–98). Though most of the EmOC facilities were established in the pre-SWAp period, the facilities became functional during the SWAp as the sector programmes ensured better availability of HRH, drugs and equipment (Arifeen *et al*. 2014). Apart from overall coverage, equity in service utilization also improved considerably—the quintile ratio of births in health facilities declined from 12 (i.e. facility delivery in the richest wealth quintile being 12 times higher than the poorest quintile) to 4 during 2001–13 (NIPORT *et al*. 2003; Sultana *et al.* 2014).

#### Health outcomes

**Figure 6.**
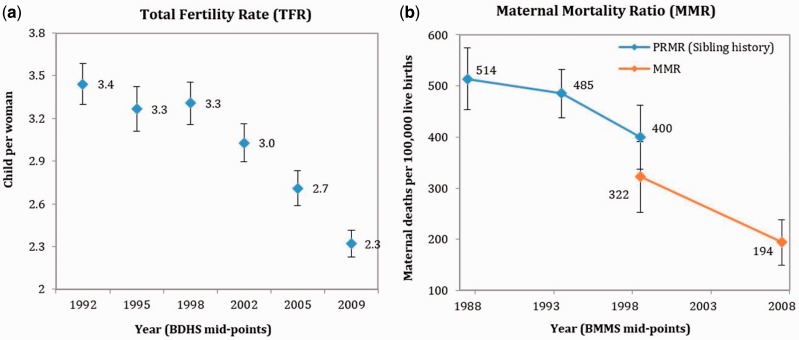
Trends in selected population and health outcomes in Bangladesh, 1992–2009

Over the last two decades, there have been impressive improvements in MCH and FP outcomes. During 1992–2009, the total fertility rate (TFR) decreased by one child per woman in rural areas, from 3.5 to 2.3 children per woman, and is approaching replacement-level fertility. Comparison between pre-SWAp and SWAp periods shows that after a decade-long plateau, the TFR resumed its decline after 1998 (see Figure 6a). The other impact indicator for the health sector, maternal mortality ratio (MMR, maternal deaths by 100 000 live births), also significantly declined during 1999–2008. Trends in pregnancy-related mortality ratio, used as a proxy for MMR, indicate that the reduction during the pre-SWAp period (i.e. 1994–99) was not statistically significant (see Figure 6b).

Outside the TFR and MMR, Bangladesh is also on track to achieve the Millennium Development Goals related to reducing child under-nutrition and mortality. Such health gains in Bangladesh were accompanied by overall socioeconomic development during the last two decades. A recent study on maternal mortality in Bangladesh identified that, apart from substantial increase in the availability of and access to health facilities after 2001, sustained improvement in factors outside the health sector such as communication, education and socioeconomic status have contributed to the improvement in maternal health through both increased use of health facilities and other pathways (Arifeen *et al*. 2014).

## Discussions

### Progress till date: what worked, and how?

#### Financial management

Following the elements of expected causal pathway to assess the impact of a SWAp (Pearson 2010), SWAps in Bangladesh’s health sector facilitated considerable progress in a) funding and technical support for programme implementation and b) design and implementation processes. Commitments from the DPs for SWAp financing increased from around US$ 800 million in HPSP to US$ 1.8 billion in HPNSDP (see Table 2), enabling the MOHFW to plan and implement essential HNP services at scale. Over the years, budget execution capacity of the MOHFW improved substantially, which spent 76% of its yearly allocation in 2004–05 (Vaillancourt 2009), increasing to 89% during 2011–14 (GOB 2014b). Improvements in FM activities during the SWAp period is reflected by the timely preparation of financial reports, the use of the government treasury system for channelling of a substantial amount of DP-financing, and the formation of an audit committee and FM task force to monitor FM actions (World Bank 2011). Continuous support from DPs, particularly DfID and the World Bank, has helped the MOHFW in building systems in FM reporting and capacity building of the MOHFW staff in FM activities. Also, a comprehensive ADP review of all OPs under the SWAp is taking place every month, enabling the MOHFW to ensure effective utilization of development financing. However, separate preparation of development and revenue budgets of the MOHFW continues to hinder efficient decision making on the allocation of resources (GOB 2011c). Reconciling the cost centers for revenue and development budgets often takes a long time to assess the financial flows.

#### Monitoring and evaluation systems

In terms of support systems, MOHFW now has greater capacities for monitoring and evaluation (M&E) compared with previous sector programmes (Daniels and Kabir 2014). The quality of the results framework (RFW) to monitor the SWAp progress improved substantially—during the 2007 APR of HNPSP (GOB 2007), no update was available for 71% of the RFW indicators (44 out of 62), which declined to 6% (2 out of 33 results indicators) in the 2012 APR of HPNSDP (GOB 2012). Also, the RFW of HPNSDP was termed as one of the World Bank’s project-wide best examples by the Independent Evaluation Group of the World Bank (IEG 2012). In order to ensure more intensive monitoring of the SWAp implementation and progress towards anticipated targets, HPNSDP introduced OP-level indicators and bi-annual programme implementation reporting. To support the MOHFW in SWAp management and monitoring activities, a PMMU was established under the Planning Wing of MOHFW. Based on the lessons learned from an earlier effort to set up a Monitoring and Evaluation Unit with the TA of erstwhile GTZ, PMMU was formed comprising a GOB part as well as TA part consisting of long-term, external specialists supported by USAID and DfID. PMMU facilitated more intensive programme monitoring by the MOHFW by producing bi-annual programme implementation reports, assisting independent reviewers to conduct APR/MTR, and providing advisory services to the senior level of MOHFW to address critical issues. The PMMU has developed an M&E Strategy and Action Plan for the health sector programme to focus on strengthening routine health information system and structured planning for nationally representative surveys during the SWAp period to routinely feed into the SWAp monitoring system.

#### Procurement and supply chain management

The procurement and supply chain management system in the HPN sector has improved substantially over the years (GOB 2013c). Under the SWAp, a centralized procurement procedure for economy of scale in bulk purchase was introduced. This resulted in a 15-fold increase in purchasing by the Central Medical Stores Depot of DGHS, taking on orders that projects would have done in the past, along with vacancy and non-linked record-keeping systems for purchasing, warehousing and distribution. This initially created delays and inefficiency in drawing benefits as expected due to lack of adequate capacity in GOB procurement planning and management (GOB 2004, 2011c). However, now the procurement processing time is faster, trained personnel are available and a web-based procurement system is in place. This has also helped minimize mis-procurements during the SWAp period. Procurement process lead time reduced from 46 months to 26 months for major medical equipment, and the numbers of procured equipment lying idle at health facilities declined from 57% to 46%. (Simed International 2013). The procurement entities in the MOHFW and the World Bank jointly worked on improving processes and steps to further reduce the total procurement lead time to under 15 months. For promoting the stewardship role of the MOHFW to ensure quality and oversee procurement process, the newly established PLMC, staffed with both MOHFW officials and TA staff, is currently conducting basic procurement training. As procurement remains one of the major contributors to enhancing the fund absorption capacity of the MOHFW, and a major area of risk for GOB, DPs like the World Bank and USAID have continued major investments in procurement systems development including capacity building.

#### Ownership and DP coordination

The MOHFW’s role on ownership and DP coordination in regard to SWAp activities improved substantially. DPs and MOHFW agreed to modifications of several controversial parts of the second SWAp, which resulted in more coherent closure of HNPSP than the first SWAp, leaving adequate time and resources available to prepare the follow on SWAp (IEG 2014). Over the years, the MOHFW ownership for SWAp implementation has improved as seen by the MOHFW’s increased leadership role in planning, administration and monitoring (Sundewall *et al.* 2006; Martin 2009; Vaillancourt 2009). Dependence on large, external review teams for APR is diminishing over time as regular implementation review reports developed by the PMMU are increasingly used for review on critical implementation and policy issues. By many accounts, the MOHFW felt a stronger sense of ownership in the design of the third Programme compared with the second Programme (World Bank 2012b; IEG 2014).

#### Health outcomes

The final component of the causal pathway to assess the impact of a SWAp (Pearson 2010) is the status of SWAp targets achieved in terms of health outcomes. In the social sector, Bangladesh has made remarkable progress in many areas during the last decade, i.e. increase in literacy and life expectancy at birth; sustaining child immunization above 90% thus contributing to the continued decline in infant and under-five mortality; and achieving a sharp decline of MMR. This has largely been possible due to targeted programmes and investments through the SWAps due to a) a strategic coordination and integration of key FP and MCH services, which contributed to maternal and child mortality reduction (Best and Saul 2012); b) strengthened support systems, which ensured timely procurement of essential drugs and equipment and increased service provision; and c) sustained investment in MCH services.

### What did not work and why?

#### Hybrid SWAp

The general definition of SWAp (Foster *et al.* 2000; Brown *et al*. 2001; Sundewall *et al.* 2006) emphasizes that all significant funding for the sector supports a single sector policy and expenditure programme, adopting common approaches across the sector. The SWAp modality in Bangladesh’s health sector can be considered as a hybrid between a projectized approach and a SWAp in the sense that it finances a specific set of MOHFW activities, agreed between the GOB and the DPs during the programme design (Kostermans and Geli 2005; World Bank 2012b). Project Aid to SWAp does not pay for staff salaries of the MOHFW, which remained largely under the GOB’s revenue budget, and focuses instead on critical systematic activities like FM, procurement, etc., to enable the MOHFW to provide essential services and achieve health goals outlined in GOB’s FYPs.

Whilst the choice of SWAp modality as practiced by MOHFW was accompanied by desired results (World Bank 2012b), this caveat allowed the MOHFW to have parallel projects and development activities implemented by non-state actors and funded and supervised directly by the DPs outside the SWAp (known as off-budget DP support). These projects and activities have contributed to achieving the results of the ongoing SWAp, but have not been formally accounted for by the MOHFW (GOB 2014b). Over the years, MOHFW has been simultaneously expanding its development expenditure by adopting new projects (some being lumpy expenditures like building new medical colleges) outside the SWAp—by the end of FY2012-13, 26 parallel projects were being financed outside HPNSDP, consuming 46% of the MOHFW’s ADP for 2012–13 (GOB 2014b).

#### Human resources and training

While the number of trained health care providers of different categories increased substantially over the years, and vacancy rates decreased, retention of physicians in rural areas has been a major problem (NIPORT *et al*. 2015). The MOHFW has been trying to address these issues by augmenting HRH at different levels but did not focus on governance issues using long-term strategy/planning (GOB 2014b). The MOHFW’s capacity building plan lacks strategic vision, as evidenced by training funds increasingly being used for 1–2 days training/workshops which has questionable contribution to capacity building. There was inappropriate and inadequate management training for LDs to efficiently lead planning and programme processes under SWAp—Civil Surgeons and Programme Managers are appointed as LDs, who lack experience in SWAp mechanism and managing OPs that often are $20–30 million in size (GOB 2013a). Lack of capacity to provide medium- to long-term trainings in the public sector also remains a major constricting factor—the training of all field-level staff in ESP during HPSP and training for Community-based Skilled Birth Attendants during HNPSP and HPNSDP did not progress as planned with only 11 Family Welfare Visitor Training Institutes (FWVTI) and a few other training institutes under MOHFW.

#### Decentralization

The overly centralized planning processes inherent to GOB systems thwarted priority institutional and policy reform initiatives related to decentralization, autonomy and LLP in the MOHFW. Also, several institutional innovations of outsourcing private agencies to provide specific support to the MOHFW during the first two SWAps failed to deliver expected results. The PSO which was meant to serve as the Programme Implementation Unit lost its support with the change in the government in 2009, and the contract was not extended beyond 2010, a year earlier than HNPSP’s completion. The Management Support Agency to oversee NGO and private agency contracting for service diversification was able to work only in a limited area as the government decided not to outsource services from CCs and union-level facilities. Finally, the Performance Monitoring Agency for commissioning non-public providers was never established (GOB 2011a; World Bank 2012b).

#### Performance-based financing

In order to leverage changes/reforms that are deemed to contribute to SWAp objectives, and to promote achievements of key health outputs, a quarter of the pooled funding administered by the World Bank was allocated under HNPSP for performance-based financing (PBF) based on the fulfilment of agreed upon indicators by the MOHFW and DPs every year (World Bank 2005b). This modality was initially not very successful in terms of achieving the targets set and the amount set aside for PBF in the first few years was not disbursed, particularly due to inadequate incentives for results and weak linkages between the agency responsible for achieving the targets and recipient of the PBF funds (Vaillancourt 2009; World Bank 2012b). Building on the experience from HNPSP, a revised PBF modality was adopted under the HPNSDP using a Disbursement for Accelerated Achievement of Results (DAAR) approach. Under this modality, the MOHFW is eligible to use a greater share of the total IDA credit from the World Bank each year to finance eligible expenditure to cover HPNSDP activities (effectively drawing down funds programmed for year five, which is US$ 71.78 million) upon attainment of agreed upon targets. The functionality of DAAR remained similar to the previous PBF modality, particularly in the sense that there has been no disbursement yet for any of the 3 years’ achievement, and there exists no direct incentive for performers. Moreover, timeliness has been a major issue as it usually takes until the first quarter of the calendar year to finalize the DAAR indicators. In particular, Year 4 DAAR indicators (covering 2014 calendar year) were not finalized by mid-August 2014 (GOB 2014b).

### Were anticipated benefits realized?

Health SWAp in Bangladesh started as a means to improving service delivery and health outcomes through better planning and coordination. It did not aim to revolutionize the service delivery mechanisms of all sources (i.e. public, NGO and private) including funding sources or change the business processes followed by the government. During the process of SWAp implementation over the last 15 years, adjustments were made both in scope and content of service delivery as well as in modes of financing. The shift from a multiple project approach to a single sector programme by the MOHFW has not only ensured government’s leadership in preparing and implementing the health programme, but also created an atmosphere for better coordination, harmonization and alignment of multiple DP funded projects and resources. The SWAp helped to focus on critical development objectives like service coverage/access and also led to efficiency gains. It has enabled the government to establish linkages between identified objectives, strategies, activities, resources and outcomes and reduced transaction cost in terms of DP engagements, programme formulation, etc. A brief assessment of progresses made in key SWAp elements (Negin 2010) is provided in Table 3.

**Table 3.** Assessment of progress in key elements in Bangladesh’s HNP SWAps, 1998–2016

SWAp elements	Progresses made	Major constraining factors
Agreed health sector plan	Since the Health and Population Strategy 1997, all the HNP SWAps in Bangladesh were driven by HNP sector plans agreed between MOHFW and DPs; and in sync with GOB’s FYPs	None (parallel projects and off-budget activities are deviations, but broadly agree with the sector plan)
Government ownership	Ownership improved substantially over time—currently the programme review process is functionally conducted by Planning Wing of MOHFW	None
Partnership between DPs and government, and among donors	Both the GOB and DPs are committed to the aid effectiveness principles as codified in the Paris Declaration, institutionalized through GOB-DP Task Groups for technical discussions by thematic areas and Local Consultative Group (LCG) meetings for GOB-DP dialogue on strategic issues	Joint Cooperation Agreement (JCA), to promote MOHFW-DP and DP-DP coordination for SWAp not in place till halfway of HPNSDP; National level (inter-ministerial) coordination mechanisms have not worked effectively
Increased funding availability and longer term commitment	DP financing commitment for SWAp duration (5 years); GOB’s sector-wide MTBF mechanism in place to project fund availability for 5 years (earlier MTBF provided projection of fund availability for 3-year period on annual rolling basis)	Increase in GOB investment for SWAp not in line with increase in national budget; DPA monitoring improved but coordination with sector activities remains wanting; MOHFW increasing budget allocation for discrete projects, mostly large infrastructure; Huge off-budget DP investment in HNP sector continues; Some DPs getting out of HNP sector (e.g. AusAID, EU, The Netherlands, GIZ); Large inflow of global funds is a new feature
Effort to streamline funding arrangemnts	Transaction cost minimized; Pooled funding between major DPs like the World Bank, Canada, Sweden, United Kingdom, and United States, complemented by parallel funding by many DPs and MOHFW itself; Strong ADP monitoring mechanism in place; Recent trend of accommodating DP fund through separate RPA arrangement	Focus on procurement of equipment and expansion of infrastructure generate stress on programme’s resource envelope; Capacity to fully utilize the treasury model is not realized yet
Institutional capacity and good governance	MOHFW now has greater capacity for all the building blocks (planning, coordination, M&E; and procurement)	FM performance affected by various factors: dearth of finance staff at all levels, absence of an appropriate training strategy and the lack of timely follow up on issues raised by the internal and external audits; HR remains a concern Stewardship and regulatory role constrained by weak legal framework and institutional inadequacies of regulatory bodies under the MOHFW
Stability of DP and government personnel	Long-term specialized support through TA are in place; A multi-year, harmonized TA plan is followed for recruiting short-term consultants	High turnover of GOB officials obstructs sustainable capacity building of the MOHFW

## Conclusion

Available evidence demonstrates that the health sector programme in Bangladesh, despite its hybrid nature, has been successful in helping to achieve the majority of the health goals and outcomes, and in strengthening overall systems for management, implementation and programme review over the past decade. Two noticeable characteristics of health SWAp in Bangladesh are a) evolving nature in response to changing requirements of the systems, and b) government’s long-term commitment to continue this process, as evident in the recent policy decision to continue with SWAp during the GOB’s seventh FYP (GOB 2015). In a continuous effort to maintain and improve functioning of the SWAp modality in Bangladesh, the following issues deserve increased focus in the coming years:

- Pursuing multisectoral approaches under SWAp to establish better linkages with other components of human development (viz. education, social protection)—many of the priority health issues of the present and future (e.g. malnutrition, non-communicable diseases) warrant stronger coordination and collaboration among multiple sectors affecting health outcomes.
- Developing a long-term, phased plan for wider health sector-wide coverage, inclusive of non-state actors. This will help address the issues like PHC coverage in urban areas and strengthen the MOHFW’s stewardship role.
- Setting up effective supervision mechanism through functionally integrated information systems—this could in turn improve results-based performance management systems to effectively leverage reforms to address longstanding critical issues like HRH, which may further contribute to SWAp goals and objectives.

The health SWAps in Bangladesh brought desired results over the last decade and the continuity of policy as well as commitment towards SWAp, irrespective of government changes, remained the same. This is one of the key factors which has contributed to the success of health SWAp in Bangladesh. The results from health SWAp in Bangladesh deserve to be shared more broadly as an example of successful adaptation of SWAp in a developing country with complex administrative structure.
